# A geographic analysis of population density thresholds in the influenza pandemic of 1918–19

**DOI:** 10.1186/1476-072X-12-9

**Published:** 2013-02-20

**Authors:** Siddharth Chandra, Eva Kassens-Noor, Goran Kuljanin, Joshua Vertalka

**Affiliations:** 1 Asian Studies Center, Michigan State University, 427 N Shaw Lane, Room 301, East Lansing, MI, 48824, USA; 2 Urban and Transport Planning in the School of Planning, Design, and Construction and Global Urban Studies Program, 552 W Circle Drive, Room 201E, East Lansing, MI, 48824, USA; 3 Department of Psychology, Psychology Building 316 Physics Room 262, East Lansing, MI, 48824, USA; 4 Department of Geography, 673 Auditorium Road, Room 116, East Lansing, MI, 48824, USA

**Keywords:** Influenza, Population loss, Population density, Thresholds, Spatial distribution, Population growth

## Abstract

**Background:**

Geographic variables play an important role in the study of epidemics. The role of one such variable, population density, in the spread of influenza is controversial. Prior studies have tested for such a role using arbitrary thresholds for population density above or below which places are hypothesized to have higher or lower mortality. The results of such studies are mixed. The objective of this study is to estimate, rather than assume, a threshold level of population density that separates low-density regions from high-density regions on the basis of population loss during an influenza pandemic. We study the case of the influenza pandemic of 1918–19 in India, where over 15 million people died in the short span of less than one year.

**Methods:**

Using data from six censuses for 199 districts of India (n=1194), the country with the largest number of deaths from the influenza of 1918–19, we use a sample-splitting method embedded within a population growth model that explicitly quantifies population loss from the pandemic to estimate a threshold level of population density that separates low-density districts from high-density districts.

**Results:**

The results demonstrate a threshold level of population density of 175 people per square mile. A concurrent finding is that districts on the low side of the threshold experienced rates of population loss (3.72%) that were lower than districts on the high side of the threshold (4.69%).

**Conclusions:**

This paper introduces a useful analytic tool to the health geographic literature. It illustrates an application of the tool to demonstrate that it can be useful for pandemic awareness and preparedness efforts. Specifically, it estimates a level of population density above which policies to socially distance, redistribute or quarantine populations are likely to be more effective than they are for areas with population densities that lie below the threshold.

## Background

Studying influenza pandemics of the past may offer valuable lessons for preparedness for the next great pandemic [1,2]. In this paper, we analyze the 1918 influenza pandemic, during which up to 100 million people worldwide and 670,000 people in the U.S.A. are estimated to have died [3-6]. India, where over 15 million perished in the short span of one year, was the single worst-affected country in terms of total mortality [7,8]. Factors affecting mortality from the pandemic included a lack of immunity to the virus, which resulted in the infection of higher-than-normal numbers of people, its highly contagious nature [9], and the prior presence of other infections such as tuberculosis or subsequent development of pneumonia as the result of infection [10,11]. Because influenza viruses spread through human contact, geography and population density in particular are potential factors for transmission and, indirectly, human mortality. The aim of this paper is to analyze the role of population density in the influenza pandemic of 1918. Because the statistics on influenza mortality for India are deeply flawed, following Davis [8], we estimate population growth trajectories allowing for a break between 1918 and 1919 to capture population loss from the disease, and use a threshold estimation method to test whether low population density districts in India experienced rates of population loss that were different from high density districts.

### Epidemiology of the influenza pandemic of 1918–19 in India

The 1918–19 influenza pandemic was one of the worst epidemics in history with an estimated global mortality between 20 and 100 million [9,12]. The pandemic occurred in two or three waves [13,14]. The first was a mild wave in the spring of 1918, followed by a second more severe wave in the following autumn that was responsible for the majority of deaths. The third wave was sporadic [15]. The influenza pandemic of 1918–19 in India shared many characteristics with the pandemic in other parts of Asia and the world. In this account, we draw heavily on the Report of the Sanitary Commissioner of India for 1918 [16] and Chandra [17]. The virus is believed to have entered India in the early months of 1918 through the port of Bombay on the west coast. This first wave was relatively mild. The same early and mild first wave was observed in other countries including Indonesia [18], England, Scotland, and Wales [19], Portugal and Spain [20], Mexico [21], and Peru [22], and cities including New York City [23] and Copenhagen [24]. It subsided by August, only to be followed by a second and far more virulent wave that peaked between September and November 1918 in various parts of India. Indonesia [17], England, Scotland and Wales [19], Portugal and Spain [20] and Mexico [21] experienced a similar pattern of timing and relative severity. Populations that were exposed to the virus early have been shown to have benefited from acquired immunity against the deadly second wave in Denmark [24], Norway [25], and Britain [26,27]. A distinctive characteristic of this epidemic was its disproportionate impact on victims aged between 15 and 35 years [6,14,15,28-30]. This phenomenon was also observed in India [16].

### Epidemics and urban responses in history

It is no coincidence that the public health community worries about new and virulent infectious diseases [31]. As recently as 2009, an episode of pandemic influenza is estimated to have claimed between 151,700 and 575,500 lives worldwide [32]. While vaccinations are frequently the first line of defense against influenza viruses, development of vaccinations for a new strain of virus may take months. In such a situation, only short-term measures including social distancing, and in extreme cases evacuations and quarantines, can protect citizens from a severe epidemic outbreak. The early introduction of social distancing measures, such as school and church closures and banning of mass gatherings, significantly reduced excess mortality during the 1918–19 influenza pandemic [33]. With similar contagious diseases, such as SARS, plague and cholera, quarantines and evacuations have been used to counter severe outbreaks. In the 15^th^ century, for example, the government of Venice combated outbreaks of plague by establishing the “Lazzaretto Vecchio” on a small island off the coast of the Piazza San Marco [34]. More recently, in the late 19^th^ and early 20^th^ centuries, New York City quarantined travelers on Swinburne and Hoffman islands to prevent the spread of cholera [35]. At about the same time, the city established the Metropolitan Board of Health to develop zoning codes to prevent overcrowding in the city and to establish standards for sanitary conditions [36]. With reference to the influenza pandemic of 1918-19 in India, the Sanitary Commissioner of India wrote: “As the striking distance of the influenza virus is probably short the obvious ideal is free ventilation and isolation of sufferers with a view to increase the air space between infected and uninfected” [16], p.66].

Over the past two decades, sudden virus outbreaks that could have led to widespread human pandemics, including H1N1 [37], SARS [38], and H5N1 [39] prompted a series of studies on non-pharmaceutical interventions [40-48], including measures to increase social distance, such as the creation of spatial barriers through quarantine [49,50], relocating populations to ‘safe’ areas [51,52], or imposing travel restrictions [53]. Evacuations, a last resort among social distancing measures, are still used; in the aftermath of the Haiti hurricane of November, 2009, and the subsequent earthquake of January 12, 2010, the government ordered the evacuation of the capital, Port-au-Prince, to prevent the spread of epidemic cholera [54]. In sum, it is widely believed that public health interventions, including social distancing measures and the controlled movement of people to either sequester those infected or as a means to lower population density below some critical threshold can significantly decrease the likelihood of a contagious disease spreading. Yet, to date, there is little if any guidance as to what such a population density threshold might be for any disease. Using the influenza pandemic of 1918 as a case, this paper presents an approach to identifying such a threshold value as a guideline for public health policy.

### Population density as a factor in influenza population loss

Studies examining the potential relationship between population density and mortality during the 1918 influenza pandemic have produced mixed results. Garrett [55] found a positive relationship between mortality rates and population densities measured on a state-wide scale in the USA. Once cities were introduced into the equation, normalizing the mortality rate of the cities with those of the states also showed a positive relationship with population density. For Nigeria, crowding contributed to comparatively higher mortality than less-crowded areas, and “there is enough evidence to support the view that large towns suffered more than small and remote villages” [56]. During the 2009–10 influenza pandemic, it was observed that H1N1 infections were sustained over longer periods of time in Taiwanese areas with higher population densities [57]. Theoretical models for influenza and other transmissible respiratory diseases consisting of agent, host, and environment interactions usually require a high host density [58].

Chowell et al. [13] also identified a link between population density and mortality for the 1918 pandemic, though it is the opposite of Garrett’s [55] findings; in Wales and England, low population density in rural areas was positively associated with mortality. On a larger county scale, however, they found no connection between population density or residential crowding and mortality or transmissibility. Supporting this stream of research, Mills et al. [59] as well as Nishiura and Chowell [60] could not identify an association between mortality and population size or density, measured as household size. While a review of the literature suggests that the evidence linking higher population densities with higher mortality rates is mixed, intrinsically this relationship makes sense, because influenza viruses spread via human interactions [9]. With rapidly rising population densities around the world, the creation of mega cities, and growing international connectivity, there is, therefore, a dire need for more research on this phenomenon. Given its high population density and rapid urbanization, India is of particular interest for the study of the emergence and spread of viruses posing significant pandemic threats [61,62].

Despite the interest in the link between population density and influenza morbidity and mortality, little is known about critical turning points or population density thresholds above which the demographic cost of the pandemic may have exceeded that of low-population density areas. Previous studies on the 1918 pandemic have emphasized the size of populations, be they in cities, towns, or rural areas, by selecting arbitrary thresholds on the basis of jurisdictional sizes or reporting boundaries. In some instances the choice of threshold appears to have been made on the basis of convenience (i.e., using categories that may have been originally created by the producers of the data using some criterion other than epidemiology (see Table 1)). Therefore, there is a hitherto unfulfilled need for a deliberate exercise to determine thresholds that focus on the epidemiologic phenomenon at hand. The aim of this paper is to apply a threshold estimation method to identify a population density threshold separating high-density districts in British India from low-density districts. In estimating this threshold, we simultaneously test the hypothesis that the low-density districts so identified differed from high-density districts in terms of population loss. As the world becomes increasingly urbanized, knowledge of how to estimate critical levels of population density above which populations may be at graver risk of contracting or succumbing to influenza than populations in lower density areas can play an important role in fostering pandemic preparedness. The findings of our study contribute directly to the field of spatial epidemiology, which is concerned with “the study of spatial variation in disease risk or incidence” to assist public health decision making [63], p.328; [64].

**Table 1:** Sample of studies using population size or density thresholds

**Study**	**Area studied**	**Time period studied**	**Unit of analysis**	**Thresholds used**	**Reasons for selecting threshold**
McSweeney et al. [65]	New Zealand	October 17 - December 27, 1918	Entire country was divided into cities, large towns, small towns, and counties	Cities (> 20,000)	Not specified
Garrett [55]	United States	1918 - 1919	Individual states and 49 cities	Cities (> 100,000)	Data availability
Kolte et al. [66]	Denmark	1917 - 1921	22 counties, each divided into countryside and towns	Capitals, provincial towns, rural areas	Based on weekly reports sent to county health officials
Nishiura and Chowell [60]	Japan		Kanagawa, 199 regions	Cities (> 20,000); Large towns (5,000 - 20,000); Small towns (2,000 - 5,000); Villages (< 2,000)	McSweeney et al. [65] and 5,000 minimum population size for town status in Japan
Chowell [13]	England and Wales	June 29, 1918 - May 10, 1919	305 administrative units, 62 counties	Cities, towns, and rural areas	Urbanization not defined

## Results and discussion

Estimates from the initial set of models, described below, indicated multiple possible thresholds for population density. The point estimate of the threshold in *model (1)*, described in the methods section below, was 19,067 PPSM (people per square mile), for which only one district, Calcutta, lay above the threshold (see the table in the Appendix). The results from this model suggest that Calcutta, the most densely populated district in British India, with a population density of 35,025 PPSM, is an outlier. Therefore, Calcutta was removed from the dataset and the subsequent analyses were conducted using a dataset of 198 districts and 1,188 observations. The results for datasets containing the Calcutta outlier in the Appendix are broadly consistent with the results presented in the paper.

**Figure 1:**
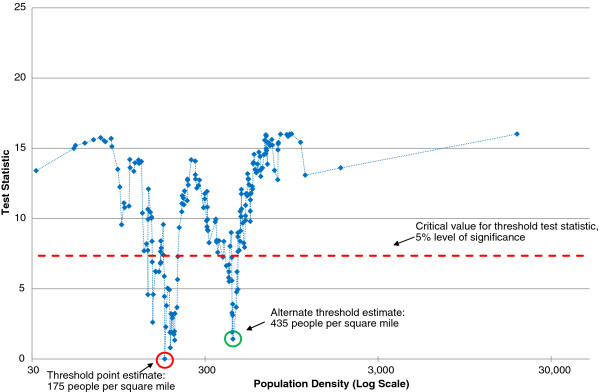
Threshold test statistic: district-specific intercepts and growth rates (Calcutta outlier dropped).

Table 2 contains the parameter estimates for the models without the Calcutta outlier. These models indicated the presence of two discrete intervals of possible threshold values. Therefore, we present two models (columns 1 and 2) corresponding to the threshold value of population density that minimized the threshold test statistic in each interval (Figure 1). Figure 1 and Column 1 in Table 2 shows the results of the threshold estimation procedure from *model (1)*. The point estimate of the threshold value of population density was 175 PPSM. At the 5% level of significance, a discontinuous set of threshold values that could not be rejected was obtained, corresponding to the intervals 148–209 and 381–464 PPSM (see Figure 1). These ranges are analogous to a 95% confidence interval. The alternate threshold estimates, presented in Column 2 in Table 2, represent values of the threshold that (a) cannot be rejected at the 5% level of significance and (b) yield the minimum threshold test statistic over the alternate range of contiguous possible (in the sense that they cannot be rejected) threshold values within which they occur. In this case, the point estimate is 435 PPSM, which produced the lowest test statistic for the 381–464 PPSM interval (see Figure 1).

**Table 2:** Threshold models for influenza population loss

**Coefficient estimate**	**Model specification: District-specific intercepts and growth rates**	
	**Threshold point estimate**	**Alternate threshold**
Time trend (β_10_)	†	†
Flu dummy (β_20_)	−0.2965***	−0.3144***
	(0.0126)	(0.0172)
Low density * flu dummy (β_30_)	0.0694***	0.0596***
	(0.0246)	(0.0221)
Time trend * flu dummy (β_40_)	†	†
Number of obs.	1188	1188
*R*^*2*^	0.9964	0.9964
	KEY DEMOGRAPHIC PHENOMENA	
Threshold population density	175	435
Range of possible thresholds (5% level of significance)	148--209	381--464
Number (percentage) of districts outside threshold range	110 (56%)	
Population loss as % of population, low density districts	−3.72%	−3.51%
Population loss as % of population, high density districts	−4.69%	−5.85%

Standard errors in parentheses.

*** p < 0.01.

†Multiple estimates, one corresponding to each district.

An important characteristic of the threshold estimation procedure is the ability to simultaneously test for differences in population loss between below-threshold and above-threshold districts. Interestingly, the difference between low-density and high-density districts is large. For the 175 PPSM threshold, below-threshold districts experienced a population growth rate of −3.72% between 1918 and 1919, while above-threshold districts experienced a growth rate of −4.69%, for a net difference of 0.97%. This difference is, moreover, statistically significant, as denoted by the significance of the parameter estimate for β_30._ For the 435 PPSM threshold, the corresponding figures are −3.51%, −5.85%, and 2.31% respectively.

In the above models, district-specific intercepts and coefficients on the time trend were also estimated. The intercepts correspond to the logarithm of district-specific population in 1891 and the coefficients on the time trend correspond to the annual rate of population growth. Because of the large number of estimates (198 each), these are not reported in Table 2.

**Figure 2:**
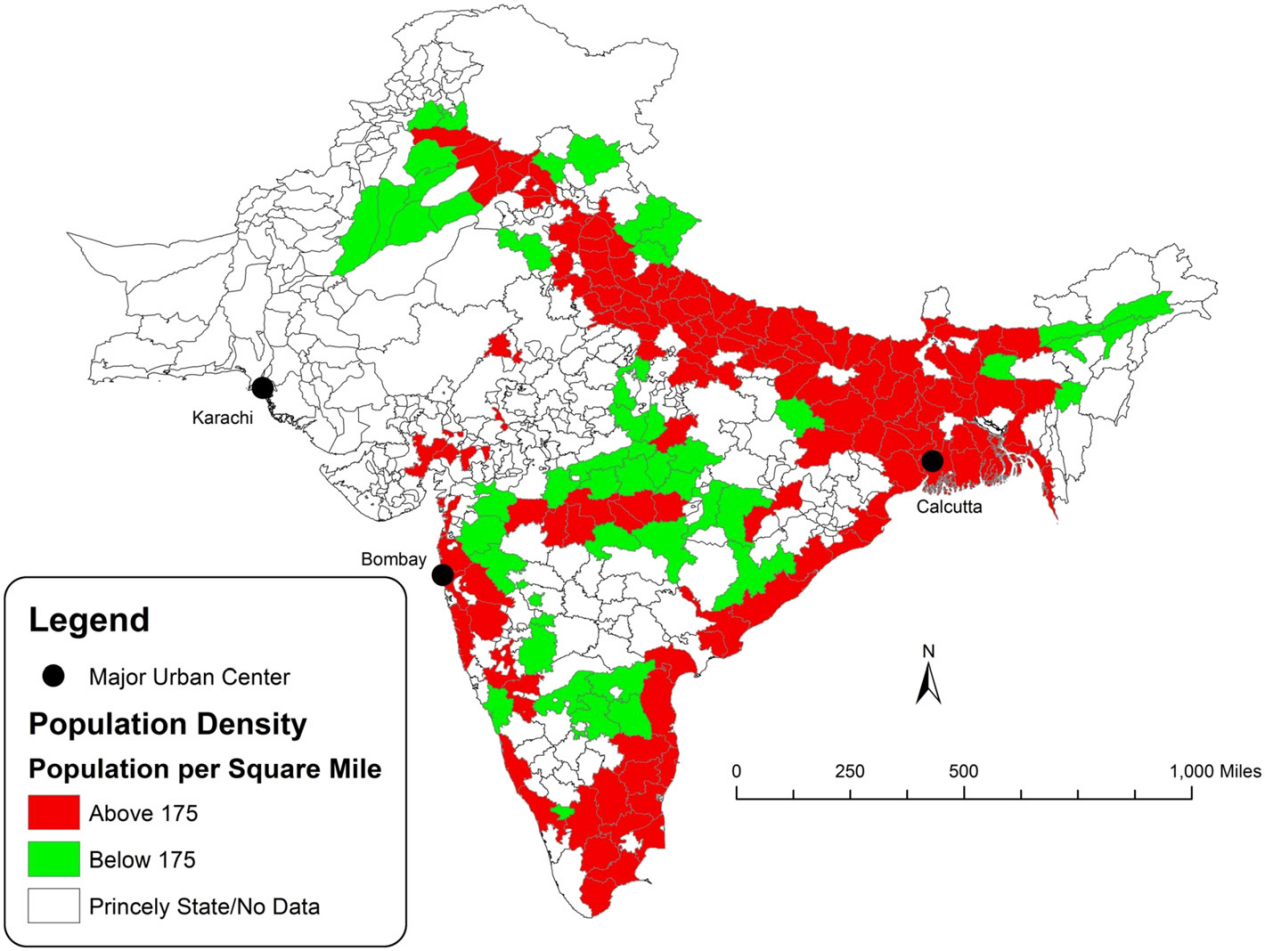
Population density threshold in India, 1918–1919.

**Figure 3:**
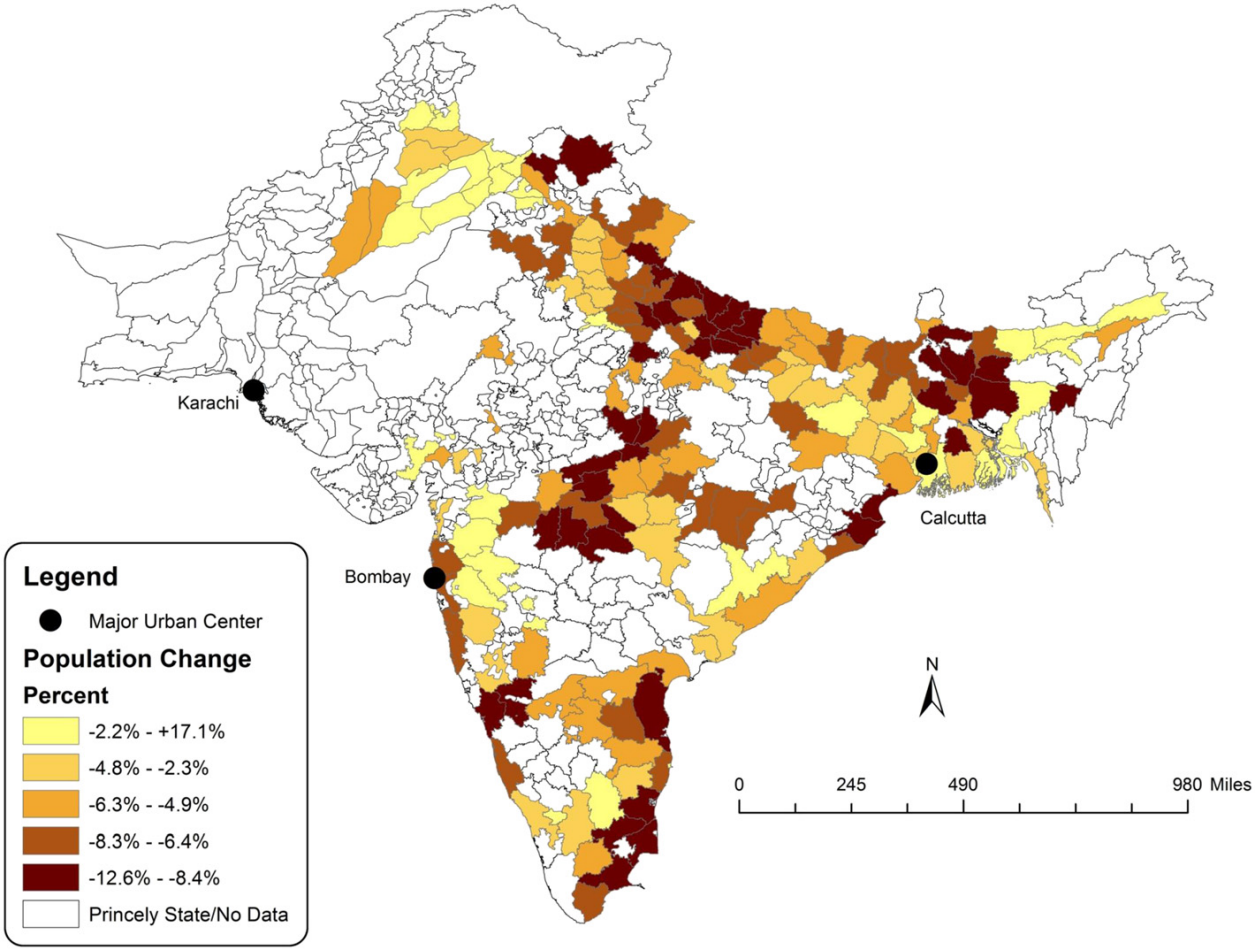
Population change in India, 1918–1919.

Figures 2 and 3 are a spatial illustration of the relationship between population density and influenza-attributable population loss. Figure 2 is a map of India that contrasts the districts that are above and below the estimated population density threshold. Figure 3 is a map of the districts arranged by quintile of population loss using estimates computed in Chandra et al. [7]. In general, the coastal areas and Gangetic plain of India show coinciding areas of above-threshold population density and high rates of population loss.

## Conclusions

The results of this study suggest the presence of population density thresholds that can be used to separate low population loss districts from high population loss districts in British India during the influenza pandemic of 1918. Using Hansen’s [67] method of threshold estimation, we identified a threshold of 175 PPSM. Below this threshold, districts experienced a decline in population of 3.72%, compared with a 4.69% decline for districts above the threshold, indicating a role for population density in understanding population loss from the epidemic. This evidence is significant in light of the often mixed findings of scholars on the relationship between population density and influenza mortality. The results from the other models presented in this paper suggest even greater differences in population loss between low- and high-density districts, so the chosen point estimates and corresponding difference in this paper should be considered conservative estimates.

### Limitations

While this study advances the literature in a number of ways, it has a number of limitations. The data do not contain information about cases of influenza or mortality that resulted directly from influenza. Therefore, the types of available data preclude analysis of case-mortality, transmission (with or without mortality), and certain other phenomena of epidemiologic significance. It is also not possible to ascribe the entire population loss from the influenza pandemic to mortality alone because of likely depressing effects of the disease on fertility in the immediate aftermath of the pandemic. Even though many studies on the 1918–1919 influenza in New Zealand, the USA and European countries have attempted to factor fertility into either post-pandemic growth rates or depression of conception during the pandemic [68-72], in the context of India, Davis [8] estimated underreporting of births [73] by as much as 50% of the true figures. While likely minimal, migration effects also cannot be accounted for due to the lack of appropriate data. In light of these limitations, we have followed the approach of Davis [8], using changes in population size between 1918 and 1919 that are not explained by the normal population growth trajectory, obtained from relatively accurate population census data, to create a picture of severity of the pandemic in the different districts of India. This paper takes the position that the estimated population loss is an indicator of the severity of the pandemic as a whole, be that loss the direct result of influenza or of other conditions resulting from influenza, including pneumonia, decreased fertility as a consequence of influenza or starvation due to pandemic-influenced famine. The data also do not contain information about socioeconomic status (i.e., poverty, social class or income, or ethnicity) [5,25,55,74,75] or remoteness [76], precluding the inclusion of other potential threshold variables that have been shown to be associated with mortality outcomes in other contexts.

The last data limitation described above also raises the issue of the constraints that the methodology places on the analysis. Thresholds may have temporal and spatial variability depending on the above variables and population structure, viral virulence, or transmissibility, though there appears to be little by way of theory or evidence on this subject. In addition, the threshold estimation method used, which has been developed relatively recently, is designed to detect a single threshold based on a single threshold variable (in this case, population density). A more versatile version of the method which allows for multiple thresholds estimated by simultaneously using multiple threshold variables would have been ideal. Finally, to the extent that the conditions in British India were different from conditions encountered in many developed and developing countries today, the results need to be interpreted in their specific context.

### Strengths

The above limitations do not detract conceptually from the utility of the sample-splitting methodology for the identification of demographic or other thresholds for health and potentially other phenomena, and for its value in establishing the critical role for population density in separating high-loss districts from districts that did not suffer as much during the single-worst epidemic of the 20^th^ century in the single-worst-affected country. In this light, this study may be viewed as a prototype on which health geographers can build using the more sophisticated data that are available in modern contexts and as the sample-splitting methodology evolves. In addition, the data used in this study cover a larger area and population than any other single-country study of the pandemic, and thus have the strength of a large sample size.

More generally, the findings presented above have a number of implications for researchers and policy makers in demography, epidemiology, planning, and public health. Most importantly, they introduce a new analytic method, threshold estimation, to the study of epidemics and their effects on populations and population growth. Subject to possible caveats about data and methodology, they also demonstrate that low population density districts in British India may not have suffered as much as high population density districts from the influenza pandemic of 1918–19. At a broader level, the mixed nature of results of studies of this question and their possible connection to contextual factors is an interesting one, and merits further study. The results of this study suggest that plans for pandemic preparedness and adaptation can be informed by the results of studies using this method, especially where high-quality data are available. Threshold estimates can be used to inform the public about location-based risk in times of such epidemics where such risk is found to be present. In addition to introducing a new analytic tool to the study of the geography of health, therefore, it is hoped that this study will be used as a template to inform guidelines for pandemic preparedness issued by public health agencies with a view to minimizing the impact of such events in the future.

## Methods

### Data sources

Following earlier studies that used census data [7,17,77], the data used in this study were obtained from six decennial censuses held in India, for the years 1891, 1901, 1911, 1921, 1931, and 1941 [78]. We focus on these censuses, and not the two censuses preceding 1891, those of 1872 and 1881, on the basis of Davis’ [8] diagnosis that the earlier censuses undercounted the population by over 1%. In addition, these two earlier censuses were conducted using methods that had significantly changed by 1891.

Within these data, we focus on population figures for the districts that were directly ruled by the British India government. We do not use parallel data from the princely states of India, which were nominally under the control of local princes and kings. Data collection by administrative authorities was in some cases significantly different from the British India administration in capacity and function. The coverage of the dataset is broad, encompassing 199 districts for each of the censuses for a total sample size of 1,194 observations. In addition, to ensure comparability over time, in the 1941 census, the population statistics for each census were reported after having been adjusted to conform to the district boundaries as of the 1941 census [78], providing a convenient dataset on population that is comparable across all the censuses. The district areas used in the computation of population density are, therefore, based on the 1941 boundaries.

### Methods

In order to estimate population density thresholds that separate low- and high-density districts on the basis of population loss from the pandemic, we use the threshold estimation technique of Hansen [67] embedded in a population growth model as follows. First, we compute the mean population density of each district across the six censuses as the population of the district divided by the area of the district in square miles to yield persons per square mile (henceforth PPSM). This mean population density provides an estimate of population density at the onset of the pandemic, and is used as the ordering variable for the threshold estimation procedure. It is also highly correlated (r = 0.999) with an alternate estimate of population density computed using the estimate of population in 1918 computed from the standard exponential population growth model allowing for a break in 1918-19 [7]. After ascertaining that the correlation between this mean population density and the dependent variable in the population growth model, namely the log of population, is not significantly different from zero (*r* = 0.036, *P* = 0.219), a condition for the threshold estimation procedure, we estimate the population density threshold. The use of the mean density rather than census-specific density ensures that, during the implementation of the sample-splitting algorithm (see below), all six observations for each district lie on the same side of the threshold.

The model used for this exercise was a fixed effects model allowing for each district to have different intercept and time trend terms to absorb heterogeneity in (the log of) population size and population growth, and allowing for a drop in the population between 1918 and 1919, the year of the pandemic. This broad approach was also employed in Davis’ [8] classic study and developed elsewhere [7,17,77]. The general model, which follows the approach of the latter three studies, can be expressed as

$$\mathrm{LPO}P_{\mathrm{it}}=\beta_{0i}+\beta_{1i}T_{t}+\beta_{2}\mathrm{FL}U_{t}+\beta_{3i}T_{t}\mathrm{FL}U_{t}+\varepsilon_{\mathrm{it}}$$

where *LPOP*_*it*_ is the log of population in district *i* in year *t*, *T*_*t*_ is a time trend, *FLU*_*t*_ is a year-specific indicator variable defined as

$$\mathrm{FL}U_{t}=\{\begin{array}{c} 1,\;t<1918\\ 0,\;t\;\geq \;1918 \end{array}$$

*ε*_*it*_ is a random error term, **β**_**0i**_, **β**_1**i**_, and **β**_3**i**_, are vectors of district-specific parameters, and *β*_2_ is a (fixed) parameter. To this model, for each of the 199 possible values of the threshold level of population density (corresponding to the 199 different districts in the sample), an indicator variable was added to the data such that the variable took on the value 1 if the observation was drawn from a district with a population density lower than the threshold and 0 otherwise. This indicator variable was interacted with the variable of interest, namely the term capturing the drop in population from the influenza pandemic to produce *model (1)*:

(1) $$\begin{array}{c} \mathrm{LPO}P_{\mathrm{it}}=\beta_{0i}+\beta_{1i}T_{t}+\beta_{2}\mathrm{FL}U_{t}+\beta_{3}I_{D}\mathrm{FL}U_{t}\\ \;+\beta_{4i}T_{t}\mathrm{FL}U_{t}+\varepsilon_{\mathrm{it}} \end{array}$$

Here, *I*_*D*_ is the indicator variable corresponding to the threshold population density *D*[67]. For each of the 199 possible values of *D*, the above equation was estimated using the 1,194 available observations. The point estimate of the threshold value of *D* was the one for which the sum of squared errors for the above model was minimized. As a robustness check of the above model, we also estimated models without district-level heterogeneity in the coefficient estimates corresponding to population growth. Thus *model (2)* was

(2) $$\begin{array}{c} \mathrm{LPO}P_{\mathrm{it}}=\beta_{0i}+\beta_{1}T_{t}+\beta_{2}\mathrm{FL}U_{t}+\beta_{3}I_{D}\mathrm{FL}U_{t}\\ \;+\beta_{4}T_{t}\mathrm{FL}U_{t}+\varepsilon_{\mathrm{it}} \end{array}$$

## Appendix: Threshold models for influenza population loss with Calcutta outlier

**Table 3:** Threshold models for influenza population loss with Calcutta outlier

			
	**Model specification: District-specific intercepts and growth rates**		
	**Threshold point estimate**	**Alternate threshold 1**	**Alternate threshold 2**
Time trend (β_10_)	†	†	†
Flu dummy (β_20_)	−0.7676***	−0.2997***	−0.3201***
	(0.1546)	(0.0128)	(0.0174)
Low density * flu dummy (β_30_)	0.4894***	0.0726***	0.0654***
	(0.1550)	(0.0250)	(0.0224)
Time trend * flu dummy (β_40_)	†	†	†
Number of obs.	1194	1194	1194
*R*^*2*^	0.9963	0.9963	0.9963
	KEY DEMOGRAPHIC PHENOMENA		
Threshold population density	19067	175	435
Range of possible thresholds (5% level of significance)	1138-19067	175-207	430-464
Number (percentage) of districts outside threshold range	52 (26%)		
Population loss as % of population, low density districts	−4.44%	−3.72%	−3.51%
Population loss as % of population, high density districts	−21.15%	−4.81%	−6.06%

Standard errors in parentheses.

*** p < 0.01.

†Multiple estimates, one corresponding to each district.

## Competing interests

None of the authors has any competing interests.

## Authors’ contributions

SC: Project leadership, design and execution of quantitative analysis; writing of entire manuscript. EK-N: Project leadership, writing of background, results, conclusion, and reference sections. GK: Design and execution of quantitative analysis; writing of analytic section. JV: Preparation of maps. All authors read and approved the final manuscript.
